# A vicious cycle of frailty and acute lower respiratory infection among community-dwelling adults (≥ 60 years): Findings from a multi-site INSPIRE cohort study, India

**DOI:** 10.1371/journal.pgph.0003903

**Published:** 2024-12-31

**Authors:** Siddhartha Saha, Ritvik Amarchand, Rakesh Kumar, Aslesh O. Prabhakaran, Prabu Rajkumar, Sumit Dutt Bhardwaj, Suman Kanungo, Radhika Gharpure, Kathryn E. Lafond, Eduardo Azziz-Baumgartner, Anand Krishnan

**Affiliations:** 1 Influenza Program, Centers for Disease Control and Prevention, New Delhi, India; 2 All India Institute of Medical Sciences, New Delhi, India; 3 National Institute of Epidemiology, Chennai, Tamil Nadu, India; 4 National Institute of Virology, Pune, Maharashtra, India; 5 National Institute of Cholera and Enteric Diseases, Kolkata, West Bengal, India; 6 Centers for Disease Control and Prevention, Atlanta, Georgia, United States of America; Chulalongkorn University College of Public Health Sciences, THAILAND

## Abstract

We studied the relationship of frailty and acute lower respiratory infection (ALRI) among a multi-site cohort of community-dwelling older adults aged ≥60 years in India. During January 2019‒January 2020, participants completed the Edmonton Frail Scale (EFS) at baseline and every 3 months at four sites in India, with each participant completing a maximum of four surveys. Participants were categorized as non-frail (0–5 points), vulnerable (6–7 points), and frail (≥8 points) based on EFS score. Project nurses made weekly home visits to identify ALRI episodes with onset during past 7 days. We estimated adjusted hazard ratios (aHR) for having an ALRI episode within 90 days after EFS by frailty category. We also assessed risk of deterioration of frailty during 7–100 days after ALRI episode onset in terms of an increased EFS score by ≥1 point and change of frailty category. Among 5801 participants (median age 65 years, 41% males), 3568 (61·5%) were non-frail, 1507 (26%) vulnerable, and 726 (12·5%) frail at enrolment. Compared with non-frail participants, the hazard of an ALRI episode was higher among vulnerable (aHR: 1·6, (95%CI 1·3–2.0) and frail participants (aHR: 1·7, 95%CI 1·3–2·2). Participants having ALRI within the past 7–100 days were at increased risk of worsening frailty category (aOR: 1.9, 95%CI 1·3–2.8) compared to participants without an ALRI episode during the same period. The association between ALRIs and worsened frailty suggests prevention of ALRIs through vaccination and other strategies may have broad reaching health benefits for older adults.

## Introduction

Frailty is a global health burden with increase in ageing population. Frailty is recognized as an age related multi-dimensional and dynamic state resulting from a cumulative decline in the functional reserve of multiple physiological systems. Frailty also makes older adults vulnerable to stressors that increase their risk of severe illness, hospitalizations, and adverse health outcomes [1].

Frailty being a state of vulnerability, its importance is increasingly felt in clinical care [2], and has been examined as a prognostic marker among older adults hospitalized with acute illnesses [3, 4]. COVID-19 has also been shown to aggravate frailty among older adults [5]. With ageing, the prevalence of multiple chronic diseases like, cardiovascular diseases, respiratory diseases, hypertension, diabetes etc. as well as disabilities also increase and together they increase the susceptibility to frailty [6, 7]. Frail older adults deteriorate further with any stressor event like severe illness, hospitalization, and falls further aggravating their health condition and making them frail [2]. Therefore, healthy ageing is imperative to optimize individual quality of life and limit the potential additional burden on health and social systems especially in low- and middle-income countries (LMICs).

Previous studies have identified frailty as a risk factor for pneumonia, hospitalization and recovery from an episode of pneumonia particularly if it is associated with influenza among older adults [8–10]. ALRI are a major cause of morbidity and mortality among older adults [11], and can be associated with post-illness functional decline [12]. Globally, influenza-associated ALRI has been estimated to cause 2·8 million episodes of hospitalization and around 2,50,000 deaths in adults aged ≥65 years annually [13, 14]. Progress towards reductions in all-cause ALRI has varied by country and has been slower among older adults than among children [11]. In India and other LMICs, where the population of adults aged ≥60 years is rapidly increasing, there are few data about the incidence and health effects of ALRI in older adults. In one rural cohort of older adults in north India, the incidence of ALRI was found to be 248.3 (95% confidence interval(CI) = 229.3–268.8) [15].

**Fig 1:**
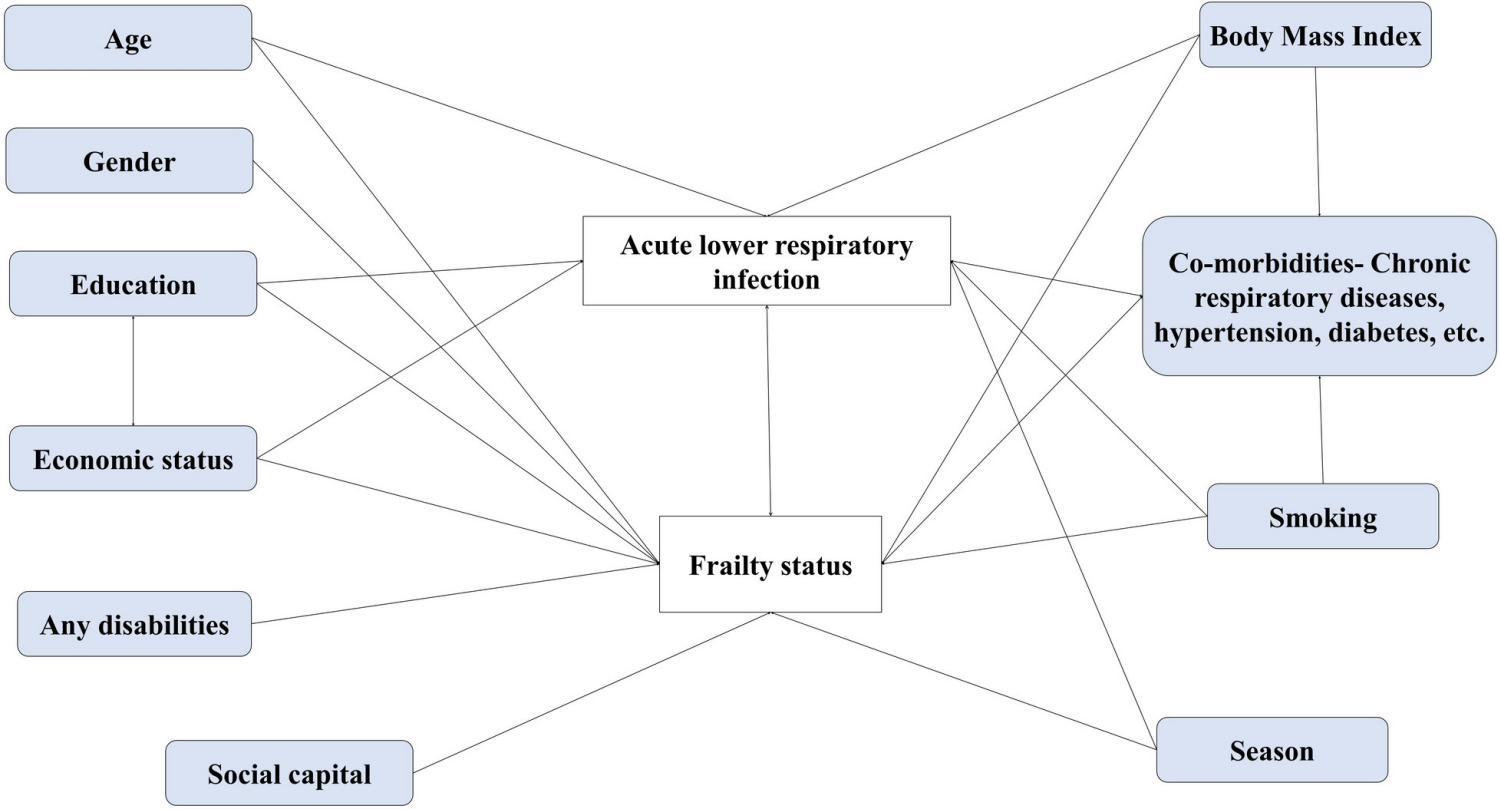
Conceptual framework to evaluate frailty as a risk factor as well as an outcome of an acute lower respiratory infection. The boxes colored white are outcome variables, viz. Acute Lower Respiratory Infection and Frailty, the gray colored boxes are other predicted variables in the conceptual framework.

In India, a middle-income country, the 2011 census identified more than 100 million adults aged ≥ 60 years and projected that almost one in five people will be aged ≥ 60 years by 2050 [3]. Although there are studies documenting prevalence of frailty among older adults [16], data remain limited about the effect of acute lower respiratory infections (ALRI) on subsequent frailty status among older adults. We hypothesized that ALRI may aggravate frailty among older adults (Fig 1). We established a multi-site cohort of community dwelling adults aged ≥60 year in India, the Indian Network of Population-Based Surveillance Platforms for Influenza and Other Respiratory Viruses among the Elderly (INSPIRE). The cohort was designed to estimate the incidence and study the predictors of ALRI [17]. We used this platform to estimate incidence rates of ALRI and evaluate frailty as a risk factor for ALRI episodes as well as ALRI as a risk factor for subsequent decline in frailty status.

## Methods

### Study design and participants

We conducted a prospective longitudinal cohort study of community dwelling older adults aged ≥ 60 years at four sites in India: Ballabgarh near Delhi in the north, Pune in the west, Chennai in the south, and Kolkata in the east from July 2018 to May 2023. The details of the INSPIRE cohort have been previously published [17]. Briefly, we enrolled community-dwelling older adults who were living at the sites for at least the past six months during July-August 2018. We enrolled annually additional participants who turned 60 years or migrated to the site and stayed for at least the past six months. We present here the data of quarterly frailty assessments and weekly ALRI surveillance from January 2019 to January 2020.

### Sample size

Based on prior published literature, an annual incidence of 0·01 episodes of ALRI among older adults, 15% prevalence of frailty among those without an ALRI episode, and odds ratio of 3.0 was considered for sample size estimation. Based on these assumptions, we got a sample size of 3,700 person-years of observation to detect deterioration of frailty status after an episode of ALRI with 95% two-sided significance and 80% power [15, 17–19].

### Data collection

We conducted quarterly four quarterly frailty assessment surveys (FAS) of participants using the Edmonton Frail Scale (EFS) from January 2019 to January 2020. Each site completed one round of FAS in approximately 90–100 days. The EFS tool covered eight domains: cognition, general health status, functional independence, social support, medication use, nutrition, mood continence, and functional performance. Trained project staff visited the houses of the participants and collected data related to the EFS tool in local languages (Tamil, Hindi, Bangla, and Marathi). Translated tools were back translated and piloted to ensure its reliability and validity [17]. Caregivers were interviewed if the participants were unable to respond to questions, especially about medicines and hospitalization.

A separate team of trained project staff visited the participants weekly at their houses to screen for respiratory symptoms in the last 7 days to detect ALRI. ALRI were defined as the presence of acute respiratory tract illness with cough, at least one respiratory symptom (breathlessness, wheeze or chest pain, or tachypnoea (respiratory rate>20)), and at least one systemic feature (measured temperature of ≥38° Celsius, or reported fever with sweating, shivers, aches and pains) [17]. Project nurses collected clinical data and mid-turbinate nasal and oropharyngeal specimens from all ALRI cases for testing for influenza viruses and respiratory syncytial virus (RSV) from non-hospitalized participants with ALRI. In addition, project staff also recorded hospitalizations because of respiratory disease and classified these as episodes of ALRI based on participant or family member reported symptoms or diagnoses on the discharge slip. Staff recorded influenza testing data for hospitalizations when available, although clinical laboratory testing for influenza is not done routinely in India. Data were collected using Open Data Kit.

### Outcome

The domains of the EFS tool were scored between 0 and 2, with 0 indicating no deficit, while presence of deficit was scored 1 or 2 depending on degree of deficit, with a total possible score of 17. We summed EFS scores of participants for each domain to categorize the participants as non-frail (0–5), vulnerable (6–7) and combined the categories of mild (8–9), moderate (10–11) and severe frailty (12–17) as frail (≥ 8) [20–22].

The outcome measure for the study was change in frailty score and frailty status as assessed by EFS tool among older adults with and without an episode of ALRI. As each site took 90–100 days to complete one round of FAS, only participants with consecutive rounds of FAS were included, with status at the beginning of each round served as baseline for that participant. Other outcome of interest was the risk that frailty poses to development of an ALRI episode. We assessed the short-term risk of developing an episode of ALRI within 90 days of frailty assessment.

### Predictor variables

We also collected data about age, sex, years of education, marital status, social interactions, civic engagements, interpersonal trust [23], household ownership and possessions, history of smoking or currently smoking, weight, height, self-reported chronic medical conditions, and self-reported disability [17]. We collected data about height using a stadiometer at enrollment and weight using portable digital weighing scales. Model covariates were identified as potential confounders in the relationship between ALRI and frailty status based on previously published studies [15, 24, 25].

As almost half of the participants were aged less than 65 years at enrolment, they were grouped into 60–64 years, 65–70 years and ≥ 70 years. We categorized level of education based on years of formal education into 0–4 years (less than primary), 5–11 years (less than high school), and ≥12(high school and above) years. We have categorized marital status as single combining ‘never married’, ‘widow/widower’ and ‘divorced/separated’ while married included ‘married’ and ‘live-in partners’. The participants were categorized into wealth quartiles using principal component analysis using combined four-site data on ownership of house and other properties, type of house, household possessions, and other amenities. Similarly, we also categorized the participants into social capital quartiles using principal component analysis based on self-reported social interactions, civic engagements, and interpersonal trust [23]. We categorized the body mass index (BMI) as underweight (<18·5), normal weight (18·5–24·9), overweight (25·0–29·9) and obese (≥30) [26]. We excluded participants for BMI calculation if they were unable to stand against the stadiometer because of illness or disability. Any comorbidity included one or more among self-reported chronic disease viz. hypertension, chronic heart disease, chronic respiratory disease, past history of tuberculosis, chronic kidney disease, diabetes, chronic liver disease, malignancy, anemia and depression. Similarly, any disability was defined as any physical movement, auditory and visual disabilities.

### Data analyses

Data were analyzed using STATA 16·1 SE (STATA Corp, Texas, USA). We used chi-squared tests to compare the distribution of participants by age group, sex, and frailty status at enrolment. Using survival analysis, we estimated incidence rates (IR) of ALRI episodes per 1000 person-years for each 90-day period following the last quarterly FAS. We analyzed the frailty status at each FAS as a risk factor for ALRI within the next 90 days among participants with at least one FAS. We used a multivariable Prentice, Williams, and Peterson Total Time (PWPTT) multiple failures survival model with days as the time scale to estimate risk of one or more episodes of ALRI [27]. We used PWPTT model to factor in the increased risk due to an episode of ALRI on a subsequent episode. We adjusted for age group, sex, years of education, wealth quartile, social capital quartile, history of smoking, BMI categories, self-reported chronic disease, period of FAS in calendar quarters, interval between FAS and onset of ALRI episode and study site as they were either significant or had p-value<0.25 in the univariate analysis. We checked for collinearity among these predictor variables and checked the regression model using post-estimation methods.

We also assessed risk of increased frailty between 7 to 100 days after onset of ALRI, both as increase in EFS score and change to worse frailty category (non-frail to vulnerable/frail or vulnerable to frail). As the surveillance was conducted weekly, the minimum interval was kept at 7 days, and maximum duration was 100 days to include the maximum interval between two consecutive FAS rounds. We measured the change in EFS score of participants who had at least two consecutive rounds of FAS and analyzed these as quarterly panel data. We used random effects multivariable logistic regression to estimate the adjusted odds ratio of increase in the EFS score by at least 1 point among those who did and did not have ALRI in last 7–100 days. As the frailty survey was conducted four times in a year on the same group of participants, the random effects in the model were used to account for multiple repeated measures. Similarly, we analyzed worsening frailty category as a potential outcome attributable to occurrence of an episode of ALRI in the last 7–100. Based on prior published literature, in the multivariable model, in addition to the variables included in the model examining frailty as a risk factor for ALRI, we included interval (in days) between the onset of acute lower respiratory tract infection and frailty assessment date, calendar quarter of survey, and detection of influenza [2, 28]. We included variables in the multivariable model that were significant or had p-value<0.25 in the unadjusted analysis. All analysis was rechecked by a statistician at CDC, Atlanta independently.

### Ethics

The study was cleared by the institutional ethical committees of all the participating institutions (AIIMS -IEC-283/02-06-17, NIE-IHEC/2017-03, NIV-IEC/2018/D5, NICED-A-1/2017-IEC) and annually reviewed. The ethical review board of the US CDC relied on the review of the AIIMS, New Delhi and IRB of other three participating institutions (Protocol No:7145). Project staff read out the information sheet designed in local language and took written consent from all participants from 1^st^ May 2018 at the time of enrolment in presence of family member witness; for participants who were unable to sign, left thumb impression was taken.

## Results

### Study cohort

During January 2019 to January 2020, we assessed the frailty status of 5,801 adults aged ≥ 60 years at least once, 4,946 (85·3%) twice, 4,721 (81·3%) thrice and 4,193 (72.3%) four times. There were 215 (3·7%) participants who migrated out of the study area and 214 (3·7%) deaths during the study period (S1 Fig)

The median age of the enrolled participants was 65 years (interquartile range (IQR): 62–70 years) and more than half of the participants were females (58·7%). While the sex distribution of participants was similar across the sites (S1 Table), those in Ballabgarh were older (median = 67 years, inter-quartile range (IQR) = 64–73 years) compared to other sites, (p <0·001). More than two-thirds of the participants in Ballabgarh, Kolkata and Pune had less than five years of formal education, while in Chennai 36·4% had less than five years of education (p <0·01). Economically, 60·8% of participants in Ballabgarh were in the highest wealth quartile compared to only 1% of participants in Kolkata (p <0·01).

### Prevalence of frailty

At enrolment, 12·5% of the 5801 participants were categorized as frail (EFS score > = 8), 26·0% as vulnerable (EFS score 6–7), and 61·5% as non-frail (EFS score 0–5) (Table 1). Women were more likely to be vulnerable or frail than men (p <0·01). Similarly, participants aged ≥ 70 years were more likely to be frail compared to those aged <75 years (p<0·01). Adults with >5 years of education and those belonging to the uppermost social quartile or uppermost wealth quartile were more likely to be non-frail.

**Table 1:** Baseline characteristics at enrolment by frailty categories of a multi-site community-dwelling cohort of adults aged ≥60 years (N = 5801)^a^ during 2019–20.

Frailty categories at enrolment									
	No Frailty		Vulnerable		Frail		Total		p- value^b^
	No.	(%)	No.	(%)	No.	(%)	No.	(%)	
**Overall**	3,568	61.5	1,507	26.0	726	12.5	5,801	100.0	<0.01
**Age Group**									
60-64y	1,747	67.8	602	23.4	227	8.8	2,576	100.0	<0.01
65-69y	1,073	63.8	406	24.1	203	12.1	1,682	100.0	
> = 70y	748	48.5	499	32.3	296	19.2	1,543	100.0	
**Gender**									
**Male**	1,640	68.4	524	21.9	232	9.7	2,396	100.0	<0.01
**Female**	1,928	56.6	983	28.9	494	14.5	3,405	100.0	
**Years of education**									
**0 years**	1,606	53.2	934	30.9	481	15.9	3,021	100.0	<0.01
**1–10 years**	1,237	66.8	413	22.3	201	10.9	1,851	100.0	
**> = 10 years**	725	78.0	160	17.3	44	4.7	929	100.0	
**Marital Status** ^**c**^									
**Single/others**	1,147	49.8	766	33.3	389	16.9	2,302	100.0	<0.01
**Married**	2,421	69.2	741	21.2	337	9.6	3,499	100.0	
**Social Capital**									
**Lowest quartile**	711	48.8	493	33.8	254	17.4	1,458	100.0	<0.01
**Second quartile**	1,009	59.3	473	27.8	220	12.9	1,702	100.0	
**Third quartile**	998	65.1	363	23.6	173	11.3	1,534	100.0	
**Highest quartile**	850	76.8	178	16.1	79	7.1	1,107	100.0	
**Wealth Quartiles**									
**Lowest quartile**	697	48.2	506	35.0	244	16.8	1,447	100.0	<0.01
**Second quartile**	846	59.2	397	27.8	186	13.0	1,429	100.0	
**Third quartile**	977	67.3	330	22.7	146	10.0	1,453	100.0	
**Highest quartile**	1,048	71.2	274	18.6	150	10.2	1,472	100.0	
**Ever Smoked**									
**Never smoked**	2,442	59.1	1,163	28.1	530	12.8	4,135	100.0	<0.01
**Past Smoker**	378	63.4	143	24.0	75	12.6	596	100.0	
**Current Smoker**	748	69.9	201	18.8	121	11.3	1,070	100.0	
**BMI Category**									
**Normal weight**	1,808	63.7	727	25.6	305	10.7	2,840	100.0	<0.01
**Underweight**	457	49.5	272	29.5	194	21.0	923	100.0	
**Overweight**	973	66.0	348	23.6	154	10.4	1,475	100.0	
**Obese**	324	59.2	155	28.3	68	12.5	547	100.0	
**Self-reported co-morbidity**									
**No**	3,184	63.2	1,269	25.2	588	11.7	5,041	100.0	<0.01
**Yes**	384	50.5	238	31.3	138	18.2	760	100.0	
**Any disability**									
**No**	190	80.5	35	14.8	11	4.7	236	100.0	<0.01
**Yes**	3,378	60.7	1,472	26.5	715	12.8	5,565	100.0	
**Study Site**									
**Chennai**	923	59.1	436	27.9	204	13.1	1,563	100.0	<0.01
**Delhi**	1,134	72.1	256	16.3	182	11.6	1,572	100.0	
**Kolkata**	730	46.9	569	36.6	257	16.5	1,556	100.0	
**Pune**	781	70.4	246	22.2	83	7.5	1,110	100.0	

EFS: Edmonton Frailty Score

a: Number of participants in the cohort who had at least one frailty assessment and were under surveillance during the study period.

b: Using Chi-squared test

c: Single included “Never Married” (3), Widow/widower (266) & Divorced/Separated(2); Married included married and live-in partners

**Fig 2:**
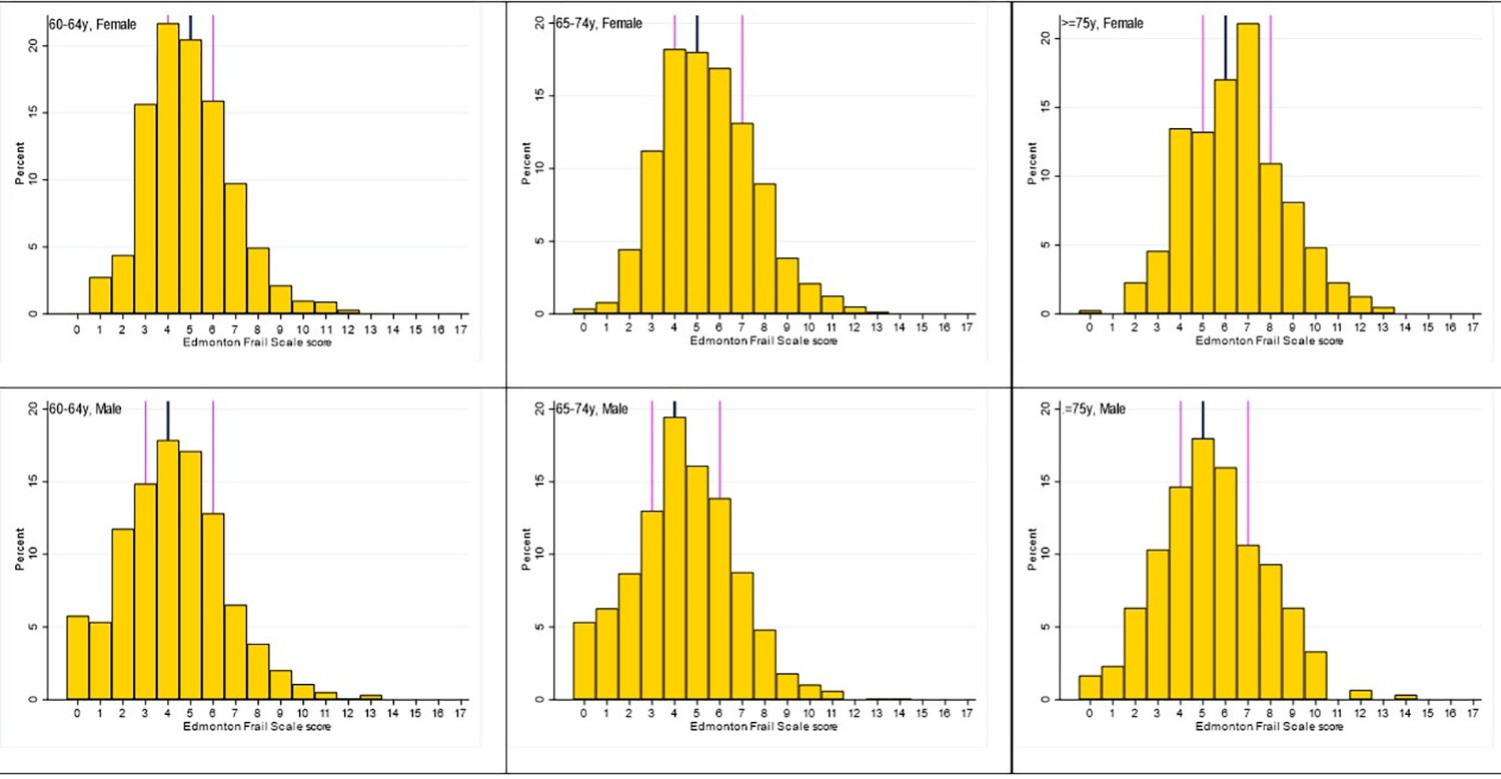
Distribution of Edmonton frail scale scores at enrolment by age-group and sex (n = 5,801). Each of the sub-graphs are grouped by sex and age-group of the participants, the vertical axis denotes the proportion of participants in numbers, horizontal axis denotes Edmonton Frail Scale(EFS) score of participants at baseline, the golden yellow bar denotes the number of participants in the respective sub-group with particular EFS score, the dark blue vertical line in center denotes median value, while the magenta lines on either side of median line denote 25^th^ and 75^th^ centiles of the respective sub-groups.

The median EFS at enrolment of female participants for all the age-groups was higher compared to males of corresponding age-groups; the medians EFS and IQR for females 60–64 years was 5 (4–6) and males 4 (3–6); females 65–74 years 5 (4–7) and males 4 (3–6); females ≥75 years 6 (5–8) and males 5 (4–7), respectively. Among the participants with all four rounds of data, the median frailty score did not change between the first round with median at 5 (4–6), round 2 at 5 (3–6), round 3 at 5 (3–6) and round 4 at 5 (3–6). Fig 2 shows the distribution of baseline frailty scores by age group and sex.

Nevertheless, frailty at individual level was observed to be dynamic with bi-directional changes occurring in EFS scores (S2 Fig). Participants categorized as non-frail during any of the FAS turned into vulnerable (1314, 14·4%, 95%CI 13·7–15·1) or frail (322, 3·5%, 95%CI 3·2–3·9) in subsequent FAS. Some vulnerable participants became frail (529, 15·6% 95%CI 14·4–16·9), while some improved to non-frail (1,496, 44·2%, 95%CI 42·5–45·9). Similarly, participants who were frail during any of the FAS could also improve to vulnerable (540, 33·5%, 95%CI 31·1–35·8) or non-frail (293, 18·1%, 95%CI 16·3–20·1) in the next FAS round.

### Frailty as predictor of ALRI

We had frailty scores and ALRI surveillance data for 5,783 participants contributing 6,888.4 person years. There were 499 ALRI episodes which occurred within 90 days after the frailty assessment used for analyzing frailty as a risk factor for ALRI. Of these 499 ALRI episodes, influenza was detected among 28 (5·7%) and RSV among 2 (0·4%). The overall incidence rates of ALRI were 109.6 (100.4–119.7) per 1000 person-years. The ALRI incidence rates were 93.7(83.3–105.5) among non-frail, 126.1 (106.6–149.2) among vulnerable, and 164.6 (133.4–203.1) among frail participants (Table 2). The participants from Ballabgarh (237.5; 95%CI: 212.9–265.0) or participants who smoked in past (216.4; 95%CI 178.9–261.8) or smoking currently (158.9; 95%CI: 134.6–187.6) had higher incidence rates of ALRI.

**Table 2:** Incidence rates of acute lower respiratory infection (per 1000-person years) by most recent frailty status among multi-site community dwelling cohort of adults aged ≥60 years (N = 5801)^a^ during 2019–20.

	Person-years	ALRI episodes	Incidence rate per 1000 person years (95% CI)
Frailty category of last survey			
**Non-frail (EFS 0–5)**	4474.4	277	93.7(83.3–105.5)
**Vulnerable (EFS 6–7)**	1632	134	126.1 (106.6–149.2)
**Frail (≥8)**	794.1	88	164.6 (133.4–203.1)
**Overall**	6900.6	499	109.6 (100.4–119.7)

EFS: Edmonton Frailty Score; ALRI: Acute lower respiratory infection

^*a*^ *Number of participants in the cohort who had at least one frailty assessment and were under surveillance during the study period*.

Univariate analysis showed age-group, years of education, marital status, higher wealth quartiles, residents of Ballabgarh, underweight, vulnerable and frail as significant co-variates. The risk of ALRI was higher among vulnerable (adjusted Hazard Ratio(aHR): 1·6(95% CI 1·3–2·0) and frail (aHR: 1·7, 95%CI 1·3–2·2) at the most recent FAS, compared to non-frail (Table 3). In addition to frailty, additional factors that were significantly associated with increased risk of ALRI in the multivariable model included history of ever smoking (aHR 1·4, 95%CI 1·1–1·7), underweight status (aHR 1·4, 95%CI 1·1–1·8), overweight status (aHR 1·3, 95%CI 1·0–1·7), obesity (aHR 1·6, 95%CI 1·2–2·2), and residing in Ballabgarh (aHR 3·6, 95%CI 2·6–4.8). Participants with 5–11 years of education (aHR 0·7, 95%CI 0·5–0·9) and those in the highest wealth quartile (aHR 0·6, 95%CI 0·4–0·8) had lower risks of ALRI.

**Table 3:** Predictors of acute lower respiratory tract infection among adults aged ≥60 years during 2019–20 (N = 5,783)^a^.

Predictors	Crude Hazards ratio	(95% CI)	Adjusted Hazards ratios	(95% CI)
**Age group**				
60-64y	Ref	(1.2–1.9)	Ref	
65-69y	1.5***	(1.5–2.4)	1.1	(0.9–1.4)
≥70y	1.9***		1.1	(0.8–1.3)
**Sex**				
Male	Ref	(1.0–1.5)	Ref	
Female	1.2**		0.9	(0.7–1.1)
**Years of education**				
Illiterate	Ref	(0.5–0.8)	Ref	
1–10 years	0.6***	(0.3–0.5)	0.8**	(0.6–1.0)
10 years or more	0.4***		0.5***	(0.3–0.7)
**Marital status** ^**b**^				
Single/others	Ref	(0.6–0.9)	Ref	
Married	0.8***		0.8*	(0.7–1.0)
**Social capital quartile**				
Lowest quartile	Ref		Ref	
Second quartile	1.4**	(1.0–1.8)	1.2	(0.9–1.6)
Third Quartile	1.7***	(1.3–2.2)	1.0	(0.7–1.3)
Highest quartile	1.7***	(1.3–2.2)	1.0	(0.7–1.3)
**Wealth quartile**				
Lowest quartile	Ref		Ref	
Second quartile	1.0	(0.7–1.3)	0.7*	(0.5–1.0)
Third quartile	1.5***	(1.1–1.9)	0.9	(0.6–1.2)
Highest quartile	1.6***	(1.3–2.1)	0.6***	(0.4–0.8)
**Sites**				
Chennai	Ref		Ref	
Ballabgarh	4.0***	(3.1–5.1)	3.8***	(2.8–5.1)
Kolkata	0.7*	(0.5–1.0)	0.4***	(0.3–0.7)
Pune	1.1	(0.8–1.6)	1.0	(0.7–1.5)
**Smoking history**				
Never smoked	Ref		Ref	
Smoked in past	2.7***	(2.2–3.4)	2.0***	(1.5–2.5)
Current smoker	2.0***	(1.6–2.5)	1.1	(0.9–1.4)
**BMI category (n = 5785)** ^**c**^				
Normal Weight	Ref		Ref	
Underweight	1.8***	(1.4–2.3)	1.5***	(1.2–1.8)
Overweight	1.1	(0.8–1.3)	1.4**	(1.1–1.7)
Obesity	1.3	(0.9–1.7)	1.6***	(1.2–2.2)
**Self-reported co-morbidity**				
No	Ref			
Yes	1.1	(0.8–1.6)		
**Frailty category at the most recent assessment survey**				
Non-Frail (0–5)	Ref		Ref	
Vulnerable (6–7)	1.3***	(1.1–1.6)	1.6***	(1.3–2.0)
Frail (8–17)	1.7***	(1.4–2.2)	1.7***	(1.3–2.1)

*** p<0.01

** p<0.05

* p<0.1

EFS: Edmonton Frailty Survey; ALRI: Acute lower respiratory infection; BMI: Body mass index

a: Total person-time of 6888.39 person years and 499 ALRI episodes, allowing for multiple events per subject.

b: Single included “Never Married” (3), Widow/widower (266) & Divorced/Separated(2); Married included married and live-in partners

c: Body mass index was not available for 16 participants.

### ALRI as a risk factor for frailty

We had EFS scores of at least two consecutive frailty assessment surveys for 4554 participants. Participants who were newly enrolled, migrated (215; 2·7%) or died (214; 2·7%) during any of the quarters and did not have consecutive rounds of frailty assessments were not included in this analysis. There were 314 (62.9%, 314/499) episodes of ALRI for which change in frailty scores could be assessed within 7–100 days of onset of ALRI. The mean EFS score within 7–100 days after ALRI episode was 5·3 (SD 2·3) compared to mean EFS score of 4·7 (SD 2·2) among participants without an ALRI during the same period. Non-frail (aHR 5.7, 95%CI 4.7–7.0) and vulnerable participants (aHR 2.1, 95%CI 1.7–2.6) were more likely to have deterioration of EFS scores after an episode of ALRI. Older age (i.e. ≥65 years) (65–74 years- aHR 1.2, 95%CI 1.1–1.4), > = 75 years–aHR 1.7, 95%CI 1.5–1.9), past history of smoking (aHR 1.2, 95%CI 1.0–1.5) and being underweight (aHR 1.3, 95%CI 1.1–1.5) increased the risk of a worsening of frailty score by at least one point compared to their last frailty assessment.

Compared to vulnerable participants, non- frail (aHR 2.0, 95%CI 1.6–2.5) were more likely to have worsened frailty category after an ALRI episode 7–100 days following the onset of ALRI. Compared to participants without ALRI episodes, a higher proportion of those with ALRI experienced subsequent worsening of their frailty category to vulnerable (627/5039, 12·4% vs. 36/175, 20·6%, p <0·00) or frail (133/5039, 2.6% vs. 14/175, 8·0%, p<0.00). Although, higher proportion of vulnerable participants with an ALRI episode moved to frail category (15/80, 18·7%) compared to without an ALRI episode (241/1814, 13·3%), it was not significant (p = 0·33). An episode of ALRI increased the risk (aHR 1.9, 95%CI 1.3–2.8) of worsened frailty category among non-frail or vulnerable participants (Table 4). The risk of change to worsened frailty category when re-assessed after 7–100 days was higher among participants aged 65–74 years (aHR 1·3, 95%CI 1·1–1.6) and ≥ 75 years (aHR 2.7, 95%CI: 2.2–3.3), underweight (aHR 1·5, 95% CI 1·2–1.9) and those with co-morbidity (aHR 2·3, 95%CI: 1·4–3.6). In addition, quarter of surveillance also seemed to increase the risk of deterioration of frailty. Participants who were educated, (1–10 years of education- aHR 0.7, 95%CI: 0.6–0.9; ≥10 years of education- aHR 0.4, 95% CI 0.3–0.5), and belonging to highest social capital quartiles (aHR 0.7, 95%CI: 0.7–0.9) were protected against worsened frailty category.

**Table 4:** Predictors of increased frailty among multi-site community dwelling cohort of adults aged ≥60 years (N = 4554)^a^ during 2019–2020.

	Increase in EFS score by 1 point				Worsening of frail category			
	Crude Odds Ratio	95% CI	Adjusted Odds Ratio	95% CI	Crude Odds Ratio	95% CI	Adjusted Odds Ratio	95% CI
**Age Group**								
60-64y	Ref		Ref		Ref		Ref	
65-74y	1.1**	(1.0–1.3)	1.2***	(1.1–1.4)	1.2*	(1.0–1.4)	1.3***	(1.1–1.6)
> = 75y	1.2***	(1.1–1.4)	1.7***	(1.5–1.9)	2.1***	(1.8–2.5)	2.7***	(2.2–3.3)
**Sex**							** **	** **
Male	Ref		Ref		Ref		Ref	** **
Females	1.1	(1.0–1.2)	1.2***	(1.1–1.4)	1.6***	(1.4–1.8)	1.3**	(1.1–1.6)
**Years of education**								
Illiterate	Ref		Ref		Ref		Ref	
1–10 years	0.9	(0.8–1.0)	0.9**	(0.8–1.0)	0.7***	(0.6–0.8)	0.7***	(0.6–0.9)
10 years or more	0.8***	(0.7–0.9)	0.7***	(0.6–0.8)	0.3***	(0.3–0.4)	0.4***	(0.3–0.5)
**Marital status** ^**b**^								
Single/others	Ref				Ref		Ref	
Married	1.0	(0.9–1.1)			0.6***	(0.5–0.6)	0.8**	(0.7–1.0)
**Social capital quartile**								
Quartile1 (lowest)	Ref				Ref		Ref	
Quartile 2	0.9	(0.8–1.0)			0.7***	(0.6–0.9)	0.8**	(0.7–1.0)
Quartile 3	1.0	(0.9–1.2)			0.8***	(0.7–0.9)	1.0	(0.8–1.2)
Quartile 4 (highest)	0.9	(0.8–1.1)			0.5***	(0.4–0.6)	0.7**	(0.6–0.9)
**Wealth quartile**								
Quartile1 (lowest)	Ref				Ref		Ref	
Quartile 2	1.0	(0.9–1.2)			0.8*	(0.7–1.0)	0.9	(0.7–1.1)
Quartile 3	1.0	(0.9–1.1)			0.7***	(0.6–0.8)	0.9	(0.7–1.2)
Quartile 4 (highest)	1.0	(0.9–1.1)			0.5***	(0.4–0.6)	0.9	(0.7–1.2)
**Sites**								
Chennai	Ref		Ref		Ref		Ref	
Ballabgarh	1.5***	(1.3–1.7)	1.3***	(1.1–1.5)	0.8*	(0.7–1.0)	0.6***	(0.5–0.8)
Kolkata	1.3***	(1.1–1.4)	1.2**	(1.0–1.4)	1.4***	(1.2–1.7)	0.9	(0.7–1.2)
Pune	1.5***	(1.3–1.7)	1.6***	(1.4–1.9)	1.4***	(1.2–1.7)	1.4**	(1.1–1.8)
**Ever smoked**								
Never smoked	Ref		Ref		Ref		Ref	
Smoked in past	1.1	(1.0–1.3)	1.2**	(1.0–1.5)	0.8	(0.7–1.1)	1.2	(0.9–1.5)
Current smoker	1.2**	(1.0–1.3)	1.1	(0.9–1.3)	0.7***	(0.6–0.8)	0.9	(0.7–1.2)
**BMI Category**								
Normal Weight	Ref		Ref		Ref		Ref	
Underweight	1.1*	(1.0–1.3)	1.3***	(1.1–1.5)	1.5***	(1.3–1.9)	1.5***	(1.2–1.9)
Overweight	1.0	(0.9–1.1)	1.0	(0.9–1.2)	0.9	(0.8–1.1)	1.0	(0.8–1.2)
Obesity	1.0	(0.8–1.2)	1.1	(0.9–1.3)	1.0	(0.8–1.2)	1.0	(0.8–1.3)
**Co-Morbidity**								
Yes	1.2	(0.8–1.7)			1.9***	(1.3–2.9)	2.3***	(1.4–3.6)
**Survey period**								
Jan-Mar 2019	Ref		Ref		Ref		Ref	
Apr-Jun 2019	1.3***	(1.1–1.4)	1.7***	(1.5–2.1)	1.7***	(1.4–2.0)	1.8***	(1.4–2.3)
Jul-Sep 2019	1.1	(1.0–1.2)	1.2***	(1.1–1.3)	1.2**	(1.0–1.4)	1.3***	(1.1–1.6)
Oct-Dec 2019	1.4***	(1.1–1.6)	1.4***	(1.2–1.8)	1.6***	(1.3–2.2)	1.6***	(1.2–2.1)
**Frailty status at the last assessment survey**								
Non-Frail (0–5)	4.3***	(3.5–5.2)	5.7***	(4.7–7.0)	1.2**	(1.0–1.5)	2.0***	(1.6–2.5)
Vulnerable (6–7)	1.8***	(1.5–2.3)	2.1***	(1.7–2.6)	Ref			** **
Frail (8–17)	Ref		Ref		--			
**Occurrence of ALRI episode**	1.0	(0.7–1.3)	1.0	(0.8–1.4)	1.7***	(1.2–2.5)	1.9***	(1.3–2.8)
**Influenza associated ALRI episode**	0.5	(0.2–1.4)	0.6	(0.3–1.4)	0.7	(0.2–3.2)	0.6	(0.2–1.7)
**Survey-surveillance interval**								
0–29 days	Ref				Ref		Ref	
30–59 days	0.8	(0.4–1.6)			0.8	(0.3–2.0)		
60–89 days	1.1	(0.6–1.9)			0.7	(0.3–1.5)		
90–100 days	1.2	(0.7–2.0)			0.9	(0.4–1.9)		

*** p<0.01

** p<0.05

* p<0.1

EFS: Edmonton Frailty Survey; ALRI: Acute lower respiratory infection; BMI: Body mass index

a: Number of cohort participants who had at least two consecutive frailty surveys were included for this analysis.

b: Single included “Never Married” (3), Widow/widower (266) & Divorced/Separated(2); Married included married and live-in partners

c: Detection of acute lower respiratory tract infection within past 7–100 days of last frailty assessment

## Discussion

We conducted this study to explore the relationship between frailty and ALRI among community dwelling adults aged 60 years and above. While frailty status in this multi-site large cohort of community dwelling adults aged ≥60 years was dynamic, ALRI tended to worsen frailty in the 100 days after illness and frailty was associated with subsequent risk for ALRI, highlighting the cyclical relationship between frailty and ALRI could lead to a downward spiral of functional loss and illness. The risk of deterioration of frailty between two consecutive FAS rounds was higher among the older adults aged ≥65 years, females, illiterates, history of smoking, underweight and those with at least one co-morbidity.

It is difficult to compare the prevalence of frailty between studies because of use of different frailty assessment tools and settings. An earlier study among a community cohort of adults aged ≥ 60 years from one of our current study sites in rural Ballabgarh in north India reported 22·3% of adults in the cohort were mild- to severely frail using the EFS tool [19]. Studies from India using the Fried Phenotype tool reported frailty prevalence of 11 to 26% in urban areas of India [29–31]. Although, the reported prevalence in our study is similar to the estimates from other higher income countries like Japan (7·4%), Thailand (13·9%), England (11·7%), Switzerland (10·3%) and the US (15%), some of these studies included participants aged 65 years or older and the prevalence of frailty among adults older than 75 years were lower compared to our study findings [28, 32–35].

We found that frailty is a risk factor for ALRI and that an episode of ALRI can also worsen frailty status, potentially leading to a cycle of functional decline and additional respiratory illnesses and further deterioration of frailty. Compared to adults aged 60–64 years, older participants (≥65 years) were at increased risk of developing an ALRI episode and deterioration of frailty between two consecutive rounds of FAS. Studies among hospitalized older adults aged ≥ 65 years have also shown that frailty hinders recovery from influenza and acute respiratory infection and can result in persistent worsening of frailty [8]. A similar relationship was also seen with COVID-19 with pre-frailty and frailty being commonly detected among hospitalized patients even after 5 months of discharge; almost two-thirds remained pre-frail and frail at 12 months after discharge [5].

Frailty therefore seems to play a complex role in both increasing the risk of ALRI as well as being an outcome of ALRI. It is therefore important to identify subpopulations of older adults who are at highest risk for frailty and subsequent ALRI to focus community-based interventions to delay such functional decline to prevent potentially deadly ALRI. Participants who were underweight, overweight or obese were at increased risk of occurrence of an ALRI episode as well as increased frailty. A similar finding was observed in a metanalysis both underweight (RR 1·5; 95% CI 1·1–1·9) as well as obesity (RR 1·4; 95%CI: 1·2–1·7) are risk factors for frailty [36]. Participants who smoked were also at increased risk of ALRI and increased frailty score. This may also explain the higher risk of ALRI and increased frailty scores among participants from Ballabgarh in our study, where more than half the participants reported to have smoked tobacco sometime in their life. Education was also protective for older adults for prevention of ALRI and frailty. Studies have shown education to have exponential type relationship with frailty [37]

We also found frailty can be reversible as seen in previous studies [38]; however, prior studies suggest that improvements occur over shorter periods (<1 year) and that the probability of recovery to pre-frail reduces with increasing duration of frailty [39]. Therefore, there is need for a life course approach to the management of frailty and healthy ageing. Older adults in Taiwan who adopted healthy lifestyle like maintaining body weight, abstinence from smoking and drinking alcohol, exercising, and diet control were better able to maintain or improve their frailty status [40]. In a two-year follow-up study in Japan, pre-frail participants who were engaged in moderate work (1–2 times a week) showed significant improvement in their frail status [41].

There are several limitations in this study. In the study population, many activities of daily living are gender dependent in India; for example, cooking, laundry, and housekeeping are typically done by females whereas managing money and transportation are more likely to be done by males. Also, in multi-generation families, if participants responded that they did not do these activities, it was deemed that they would need help and scored adversely. Many of these participants had difficulty in drawing clocks used to measure cognition in the EFS tool, especially those who were illiterate or had less than 5 years of schooling. These cultural and contextual factors could have influenced the frailty assessment. We used the EFS tool for frailty assessments every quarter to detect any change in frailty status which may be associated with ALRI, although the EFS tool may not have been designed for such frequent assessments. Similarly, we excluded participants who died during the study period and could not contribute to subsequent quarters causing survival bias. This could have lead us to underestimate the effect caused by ALRI. Lastly, frailty may change over short periods of time because of many other reasons which have a bearing on health like other illnesses or trauma, bereavement, sudden economic loss, etc., which might confound the observed association with ALRI.

Despite these limitations, our study is probably first such study which explored the effect of ALRI on frailty and thereby unraveled the potential vicious cyclical relationship between frailty and ALRI. This large multi-site study of over five thousand community dwelling older adults studied the common risk factors that can cause ALRI as well as aggravate frailty. Our study highlights the need for a comprehensive care for older adults targeting lifestyle, adequate nutrition, social support and awareness programs to prevent ALRI and frailty.

In conclusion, the study highlighted that frailty can both be a risk-factor and an outcome of ALRI among older adults. Therefore, a more wholistic public health approach is required for prevention and management of ALRI among this vulnerable population, especially among those belonging to lower socio-economically weaker sections. Reduced episodes of ALRI along with other health interventions in turn should help prevent increased frailty among older adults. Future studies should evaluate interventions to prevent frailty and vaccine preventable diseases because of influenza, COVID-19, RSV, and pneumococcus to reduce the hazard of ALRI and subsequent functional decline. The investments made in health by LMICs countries have resulted in increased life-expectancy. There is therefore need for policies for older adults in LMICs to ensure healthy aging to be able to reap the dividends of this increased life expectancy.

## Supporting information

S1 Fig Study profile with number of participants, new enrolments, migrations and deaths during each calendar quarter of the study. ALRI: Acute lower respiratory infection.
(DOCX)

S2 Fig Distribution of change in EFS scores between baseline and last (fourth) frailty assessment survey rounds by baseline status of the participants. Each sub-graph depicts the distribution of change in EFS score between baseline and last FAS round among participants (in percent), grouped by their baseline frailty status- Non-frail (EFS score 0–5), Vulnerable(EFS score 6–7) and Frail (EFS Score 8–17). An increase in score indicates worsened frailty and a decrease in score indicates improved frailty.
(DOCX)

S1 Table Demographic characteristics at enrolment by study site of a multi-site community dwelling cohort of adults aged > = 60 years (N = 5801)* during 2019–20. * Number of participants in the cohort who had at least one frailty assessment during the study period.**Single included “Never Married” (3), Widow/widower (266) & Divorced/Separated (2); Other responses included “Never married”(3) & “Do not want to tell”(5).
(DOCX)
