# Supplementary material for: A vicious cycle of frailty and acute lower respiratory infection among community-dwelling adults (≥ 60 years): Findings from a multi-site INSPIRE cohort study, India
Source: PLOS Glob Public Health. 2024 Dec 31;4(12):e0003903. doi: 10.1371/journal.pgph.0003903 (PMC11687819; doi:10.1371/journal.pgph.0003903)
Supplement: S1 Fig — ALRI: Acute lower respiratory infection. (DOCX) [file pgph.0003903.s001.docx]

**S1 Fig. Study profile with number of participants, new enrolments, migrations and deaths during each calendar quarter of the study.**

| **January -March 2019** | | **# of participants** |
| --- | --- | --- |
| **Weekly ALRI Surveillance** | | **5117** |
| **Quarterly Frailty survey** | | **4957** |
| **Died** |  | **33** |
| **Migrated** |  | **22** |
| **New enrolments** | | **0** |
|  |  |  |
| **April - June 2019** | | **# of participants** |
| **Weekly ALRI Surveillance** | | 5066 |
| **Quarterly Frailty survey** | | 4852 |
| **Died** |  | 47 |
| **Migrated** |  | 33 |
| **New enrolments** | | 107 |
|  |  |  |
| **July -September 2019** | | **# of participants** |
| **Weekly ALRI Surveillance** | | 5675 |
| **Quarterly Frailty survey** | | 4457 |
| **Died** |  | 38 |
| **Migrated** |  | 52 |
| **New enrolments** | | 566 |
|  |  |  |
| **October - December 2019** | | **# of participants** |
| **Weekly ALRI Surveillance** | | 5585 |
| **Quarterly Frailty survey** | | 4841 |
| **Died** |  | 52 |
| **Migrated** |  | 61 |
| **New enrolments** | | 122 |
|  |  |  |
| **January-2020** |  | **# of participants** |
| **Weekly ALRI Surveillance** | | 5452 |
| **Quarterly Frailty survey** | | 738 |
| **Died** |  | 44 |
| **Migrated** |  | 47 |
| **New enrolments** | | 48 |
|  |  |  |
| **Total participants** | | **5801** |
| **Total deaths** | | **214** |
| **Total migrations** | | **215** |

*ALRI: Acute lower respiratory infection*
