# Supplementary material for: A vicious cycle of frailty and acute lower respiratory infection among community-dwelling adults (≥ 60 years): Findings from a multi-site INSPIRE cohort study, India
Source: PLOS Glob Public Health. 2024 Dec 31;4(12):e0003903. doi: 10.1371/journal.pgph.0003903 (PMC11687819; doi:10.1371/journal.pgph.0003903)
Supplement: S2 Fig — Each sub-graph depicts the distribution of change in EFS score between baseline and last FAS round among participants (in percent), grouped by their baseline frailty status- Non-frail (EFS score 0–5), Vulnerable(EFS score 6–7) and Frail (EFS Score 8–17). An increase in score indicates worsened frailty and a decrease in score indicates improved frailty. (DOCX) [file pgph.0003903.s002.docx]

**S2 Fig. Distribution of change in EFS scores between baseline and last (fourth) frailty assessment survey rounds by baseline status of the participants**

*Legend for figure 2: Each sub-graph depicts the distribution of change in EFS score between baseline and last FAS round among participants (in percent), grouped by their baseline frailty status- Non-frail (EFS score 0-5), Vulnerable(EFS score 6-7) and Frail (EFS Score 8-17). An increase in score indicates worsened frailty and a decrease in score indicates improved frailty*

Decrease in frailty score 🡨🡪 Increase in frailty scores
