# Supplementary material for: A vicious cycle of frailty and acute lower respiratory infection among community-dwelling adults (≥ 60 years): Findings from a multi-site INSPIRE cohort study, India
Source: PLOS Glob Public Health. 2024 Dec 31;4(12):e0003903. doi: 10.1371/journal.pgph.0003903 (PMC11687819; doi:10.1371/journal.pgph.0003903)
Supplement: S1 Table — * Number of participants in the cohort who had at least one frailty assessment during the study period.**Single included “Never Married” (3), Widow/widower (266) & Divorced/Separated (2); Other responses included “Never married”(3) & “Do not want to tell”(5). (DOCX) [file pgph.0003903.s003.docx]

**S1 Table. Demographic characteristics at enrolment by study site of a multi-site community dwelling cohort of adults aged >=60 years (N=5801)* during 2019-20**

|  | **Chennai** |  | **Delhi** |  | **Kolkata** | | **Pune** |  | **Total** |  | **p-value** |
| --- | --- | --- | --- | --- | --- | --- | --- | --- | --- | --- | --- |
|  | (n=1563) |  | (n=1572) | | (n=1556) | | (n=1110) | | (n=5801) | |  |
| **Age Group** |  |  |  |  |  |  |  |  |  |  |  |
| **60-64y** | 660 | 42.2 | 496 | 31.6 | 796 | 51.2 | 624 | 56.2 | 2576 | 44.4 | <0.001 |
| **65-69y** | 425 | 27.2 | 516 | 32.8 | 456 | 29.3 | 285 | 25.7 | 1682 | 29.0 |  |
| **>=70y** | 478 | 30.6 | 560 | 35.6 | 304 | 19.5 | 201 | 18.1 | 1543 | 26.6 |  |
| **Gender** |  |  |  |  |  |  |  |  |  |  |  |
| **Male** | 678 | 43.4 | 660 | 42 | 628 | 40.4 | 430 | 38.7 | 2396 | 41.3 | 0.084 |
| **Female** | 885 | 56.6 | 912 | 58 | 928 | 59.6 | 680 | 61.3 | 3405 | 58.7 |  |
| **Years of education** |  |  |  |  |  |  |  |  |  |  |  |
| **Illiterate** | 396 | 25.3 | 971 | 61.8 | 1056 | 67.9 | 598 | 53.9 | 3021 | 52.1 | <0.001 |
| **1-10 years** | 670 | 42.9 | 404 | 25.7 | 353 | 22.7 | 424 | 38.2 | 1851 | 31.9 |  |
| **>=10 years** | 497 | 31.8 | 197 | 12.5 | 147 | 9.4 | 88 | 7.9 | 929 | 16.0 |  |
| **Marital status** |  |  |  |  |  |  |  |  |  |  |  |
| **Single/others** | 548 | 35.1 | 561 | 35.7 | 757 | 48.7 | 436 | 39.3 | 2302 | 39.7 | <0.001 |
| **Married** | 1015 | 64.9 | 1011 | 64.3 | 799 | 51.3 | 674 | 60.7 | 3499 | 60.3 |  |
| **Social Capital Quartile** |  |  |  |  |  |  |  |  |  |  |  |
| **Lowest quartile** | 245 | 15.7 | 120 | 7.6 | 557 | 35.8 | 561 | 50.5 | 1483 | 25.6 | <0.001 |
| **Second quartile** | 382 | 24.4 | 224 | 14.2 | 462 | 29.7 | 334 | 30.1 | 1402 | 24.2 |  |
| **Third Quartile** | 451 | 28.9 | 505 | 32.1 | 337 | 21.7 | 148 | 13.3 | 1441 | 24.8 |  |
| **Highest quartile** | 485 | 31.0 | 723 | 46.0 | 200 | 12.9 | 67 | 6.0 | 1475 | 25.4 |  |
| **Wealth Quartiles** |  |  |  |  |  |  |  |  |  |  |  |
| **Lowest quartile** | 138 | 8.8 | 121 | 7.7 | 1100 | 70.7 | 88 | 7.9 | 1447 | 24.9 | <0.001 |
| **Second quartile** | 301 | 19.3 | 165 | 10.5 | 327 | 21.0 | 636 | 57.3 | 1429 | 24.6 |  |
| **Third Quartile** | 630 | 40.3 | 330 | 21.0 | 113 | 7.3 | 380 | 34.2 | 1453 | 25.0 |  |
| **Highest quartile** | 494 | 31.6 | 956 | 60.8 | 16 | 1.0 | 6 | 0.5 | 1472 | 25.4 |  |
| **Smoking history** |  |  |  |  |  |  |  |  |  |  |  |
| **Never smoked** | 1223 | 78.2 | 636 | 40.5 | 1239 | 79.6 | 1037 | 93.4 | 4135 | 71.3 | <0.001 |
| **Past Smoker** | 213 | 13.6 | 261 | 16.6 | 107 | 6.9 | 15 | 1.4 | 596 | 10.3 |  |
| **Current Smoker** | 127 | 8.1 | 675 | 42.9 | 210 | 13.5 | 58 | 5.2 | 1070 | 18.4 |  |
| **BMI Category** |  |  |  |  |  |  |  |  |  |  |  |
| **Normal weight** | 685 | 43.9 | 830 | 52.8 | 742 | 47.8 | 583 | 52.9 | 2840 | 49.0 | <0.001 |
| **Underweight** | 170 | 10.9 | 321 | 20.4 | 291 | 18.8 | 141 | 12.8 | 923 | 15.9 |  |
| **Overweight** | 511 | 32.8 | 315 | 20.0 | 366 | 23.6 | 283 | 25.7 | 1475 | 25.4 |  |
| **Obese** | 193 | 12.4 | 106 | 6.7 | 152 | 9.8 | 96 | 8.7 | 547 | 9.4 |  |
| **Self-reported co-morbidity** | |  |  |  |  |  |  |  |  |  |  |
| **No** | 1290 | 82.5 | 1440 | 91.6 | 1290 | 82.9 | 1021 | 92.0 | 5041 | 86.9 | <0.001 |
| **Yes** | 273 | 17.5 | 132 | 8.4 | 266 | 17.1 | 89 | 8.0 | 760 | 13.1 |  |
| **Any disability** |  |  |  |  |  |  |  |  |  |  |  |
| **No** | 58 | 3.7 | 27 | 1.7 | 61 | 3.9 | 90 | 8.1 | 236 | 4.1 | <0.001 |
| **Yes** | 1505 | 96.3 | 1545 | 98.3 | 1495 | 96.1 | 1020 | 91.9 | 5565 | 95.9 |  |
| **EFS category at enrolment** | |  |  |  |  |  |  |  |  |  |  |
| **No Frailty** | 923 | 59.1 | 1134 | 72.1 | 730 | 46.9 | 781 | 70.4 | 3568 | 61.5 | <0.001 |
| **Vulnerable** | 436 | 27.9 | 256 | 16.3 | 569 | 36.6 | 246 | 22.2 | 1507 | 26.0 |  |
| **Frail** | 204 | 13.1 | 182 | 11.6 | 257 | 16.5 | 83 | 7.5 | 726 | 12.5 |  |

** Number of participants in the cohort who had at least one frailty assessment during the study period.**Single included “Never Married” (3), Widow/widower (266) & Divorced/Separated (2) ; Other responses included “Never married”(3) & “Do not want to tell”(5)*
